# Selection Mapping Identifies Loci Underpinning Autumn Dormancy in Alfalfa (*Medicago sativa*)

**DOI:** 10.1534/g3.117.300099

**Published:** 2017-12-18

**Authors:** Gitanshu Munjal, Jingjie Hao, Larry R. Teuber, E. Charles Brummer

**Affiliations:** * Department of Plant Sciences, University of California-Davis, California 95616; † Plant Breeding Center, University of California-Davis, California 95616

**Keywords:** selection, mapping, alfalfa, dormancy

## Abstract

Autumn dormancy in alfalfa (*Medicago sativa*) is associated with agronomically important traits including regrowth rate, maturity, and winter survival. Historical recurrent selection experiments have been able to manipulate the dormancy response. We hypothesized that artificial selection for dormancy phenotypes in these experiments had altered allele frequencies of dormancy-related genes. Here, we follow this hypothesis and analyze allele frequency changes using genome-wide polymorphisms in the pre- and postselection populations from one historical selection experiment. We screened the nondormant cultivar CUF 101 and populations developed by three cycles of recurrent phenotypic selection for taller and shorter plants in autumn with markers derived from genotyping-by-sequencing (GBS). We validated the robustness of our GBS-derived allele frequency estimates using an empirical approach. Our results suggest that selection mapping is a powerful means of identifying genomic regions associated with traits, and that it can be exploited to provide regions on which to focus further mapping and cloning projects.

Alfalfa (*Medicago sativa*) is a highly nutritious forage used as hay, silage, and pasture for dairy and beef cattle. The major trait used to classify alfalfa germplasm is the expression of autumn dormancy (or “fall dormancy”), a plant’s response to decreasing photoperiod and temperature (and possibly light quality) in autumn (Castonguay *et al.* 2006). The onset of dormancy in autumn occurs during a period of acclimation that eventually concludes with the expression of cold and desiccation tolerance (Thomashow 1999; McKenzie *et al.* 1988). Further, these changes in metabolism are associated with the slowing and eventual complete cessation of growth during the dormant period. Dormant germplasm exhibits reduced growth and an increased decumbent shoot orientation in autumn compared with nondormant germplasm, which continues to grow upright. This process allows the dormant plants to survive stress until favorable conditions for growth return. Such seasonal rhythms of dormancy are observed in both woody and herbaceous perennial species (Thomashow 1999).

Autumn dormancy in alfalfa is under complex quantitative genetic control. However, results from inheritance studies indicate that additive components of the genetic variance of the trait are generally of more importance than the nonadditive components (Daday and Greenham 1960; Castonguay *et al.* 2006). This suggests the potential for selection programs to effectively make gains for the trait in variable populations, as has indeed been successfully implemented (*e.g.*, Cunningham *et al.* 1998). Across multiple populations, phenotypic selection for increased or decreased autumn plant height resulted in either more or less winter injury, respectively (Cunningham *et al.* 1998); conversely, selection for decreased winter injury resulted in a decrease in autumn plant height (Weishaar *et al.* 2005). The relationship between the traits, when evaluated across germplasm spanning the entire range of dormancy levels, is strong (Schwab *et al.* 1996); however, the relationship when evaluated on germplasm with a smaller dormancy range is minimal (Brummer *et al.* 2000, and references therein). These results suggest the feasibility of manipulating fall growth without affecting winter survival at least to a degree. Further, this opportunity may be realized quickly if genomic regions controlling autumn dormancy could be targeted by marker-assisted selection (Li *et al.* 2015; Brouwer *et al.* 2000). In addition to winter survival, fall dormancy is also associated with other important agronomic traits, including spring regrowth, regrowth rate after harvest throughout the year, forage nutritive value, plant maturity, and biomass yield (Levitt 1956; Smith 1961; Busbice and Wilsie 1965). Thus, understanding autumn dormancy could further our understanding of the genetic basis of these other traits as well.

Selection on genetically determined traits alters the frequency of trait-associated variant alleles in a population. Characterizing genomic changes that occurred in a population as a result of selection, *e.g.*, changes in gene frequencies (Lewontin and Krakauer 1973) and/or in the amount of genetic variation (Tajima 1989), is a useful technique to identify loci associated with the trait. Further, mapping in experimental populations developed by recurrent phenotypic selection has the considerable advantage over other mapping approaches—*e.g.*, quantitative trait locus mapping in biparental populations and genome-wide association studies (GWAS) in diversity panels—of encompassing a large amount of genetic variation while also enabling the detection of initially rare alleles that accumulate over generations (Wisser *et al.* 2011). Such “selection mapping” has been used to study the genetic control of diverse traits in various organisms such as maize (*Zea mays* L.) (Wisser *et al.* 2008; Beissinger *et al.* 2014), dog (*Canis lupus familiaris*) (Vaysse *et al.* 2011), and sheep (*Ovis aries*) (Gutiérrez-Gil *et al.* 2014). In recent years, selection mapping has been conducted by pooling multiple individuals from pre- and postselection populations and using next-generation sequencing to yield highly accurate population-specific allele frequency estimates, thereby reducing experimental costs [for a detailed discussion, see Schlötterer *et al.* (2015)].

Here, we hypothesize that mapping in experimental populations selected for more and less dormancy will help identify loci controlling the expression of autumn dormancy in alfalfa. Following this hypothesis, the present study uses the cultivar CUF 101 and populations derived from it by recurrent selection for more and less autumn growth in order to (1) assess phenotypic divergence between populations, (2) calculate allele frequencies of biallelic markers in each population, (3) assess the reliability of the calculated frequencies, and (4) identify genomic regions controlling autumn dormancy by analyzing allele frequency shifts during selection.

## Materials and Methods

### Plant material

The cultivar CUF 101 (Lehman *et al.* 1983) was divergently selected for three cycles for taller and shorter plants in autumn as described previously (Cunningham *et al.* 1998). Briefly, 500 plants from the original population were evaluated for autumn dormancy by measuring regrowth height following autumn clipping. The 45 shortest and the 45 tallest plants were selected as the parents for the more- and the less-dormant cycle 1 populations, respectively. The selected parents were intercrossed in the greenhouse without emasculation, and the resulting seed was used for the next cycle of selection. Three cycles of selection, maintaining the same population size, were completed from the initial cycle 0 population. For this experiment, we analyzed the initial CUF 101 population and the cycle 3 shorter and taller populations, hereafter referred to as CUF-O, CUF-L, and CUF-H, respectively.

### Phenotypic data

Seeds from the three populations were planted in a greenhouse in Davis, CA, in June 2014. For each population, 96 healthy plants were randomly chosen for field evaluation and subsequently transplanted to the field embedded within a broader experiment containing other entries. The experimental design in the field was a randomized complete block design with four replications, with plots of 24 plants representing an experimental unit. Autumn regrowth data were collected in November 2014 by measuring the natural height of each plant as the distance from the soil surface to the top of the tallest stem as it stood in the field (*i.e.*, without artificially extending to full length). The raw data were standardized and fitted to a linear model treating blocks as a fixed effect. Residuals from this model were then used to estimate phenotypic distributions for each of the three populations. The variances and means of the distributions were compared at the 95% confidence level using *F*- and *t*-tests, respectively.

### Genotypic data

Two to three newly formed trifoliolate leaves were sampled individually from each of the 288 plants (96 plants per population × 3 populations). Subsequently, tissue samples were lyophilized and ground. DNA was extracted from ground tissue using DNeasy 96 plant kits (Qiagen, 69181) and quantified using Quant-iT PicoGreen dsDNA Assay Kit (Life Technologies, P7589). GBS libraries were constructed using a protocol modified from Elshire *et al.* (2011) as described previously in Li *et al.* (2014). Briefly, 100 ng of DNA from each sample was digested with ApeKI (NEB, R0643L). Two different types of adapters, a barcode adapter and a common adapter, were ligated with the DNA fragment using T4 DNA ligase (NEB, M0202L). Equal volumes of the ligation product from each sample were pooled together and purified using the QIAquick PCR Purification Kit (QIAGEN, 28104). The ligation product was then amplified with the KAPA Library Amplification Readymix (Kapa Biosystems, KK2611) using 50 ng of DNA as the template and 25 nmol of each PCR primer in a 50 μl reaction system. PCR was performed under the following thermal cycling conditions: 72° for 5 min; denaturation at 98° for 30 sec; followed by 10 cycles with 10 sec of denaturation at 98°, 30 sec of primer annealing at 65°, and 30 sec extension at 72°. The resulting library was again purified using the QIAquick kit (QIAGEN, 28104). The library was sequenced in two lanes on an Illumina Hi-Seq2500 at the University of Texas Southwestern Medical Center in Dallas, Texas.

### Allele frequency estimates

Cultivated alfalfa (*M. sativa*) lacks a reference genome, and the closest relative with a well-documented reference genome (*Medicago truncatula*) has a genome size roughly half that of *M. sativa* (Blondon *et al.* 1994). Therefore, in order to target alfalfa specific tags, we used the GBS-SNP-CROP pipeline (Melo *et al.* 2016) for *de novo* single-nucleotide polymorphism (SNP) discovery. This pipeline uses quality-filtered demultiplexed reads to build a GBS-specific reduced-representation reference (hereafter *mock reference*) by clustering reads based on a similarity threshold. SNP calling is then performed relative to the mock reference. Here, we used one lane of data in clustering. We followed the default suggestions for single-end data with a few exceptions. The similarity threshold was raised to 0.95 and the clustering step was implemented using the program *vsearch* Rognes *et al.* (2016) in order to overcome the memory limitations of the default clustering algorithm. Further, all pipeline steps following the reference development were parallelized using either the perl Parallel::ForkManager module or custom bash scripts (demo available at https://github.com/grmunjal/tutgit).

For every individual, read counts were extracted for every variable position. Read counts across all individuals from a given field plot (hereafter *sample*) were pooled *in silico* for every marker to get sample specific read counts. To retain a set of high-confidence SNPs for frequency estimation, we required every marker in every population to have at least 96 reads, equal to the number of individuals in the population. To control for copy number variation, we set the upper limit on read counts within each population equal to the mean coverage plus two standard deviation (SD) within that population. Further, using the list of nonredundant consensus sequences (hereafter *clusters*) provided by the GBS-SNP-CROP pipeline, we required every marker to originate from a cluster with valid alignments (here defined as primary alignments with mapping quality ≥30) to either the *M. truncatula* reference genome (Tang *et al.* 2014) or the recently released cultivated alfalfa at the diploid level (CADL; Bingham and McCoy 1979) assembly (v0.95; Medicago HapMap, Alfalfa Breeder’s Toolbox) using BWA-MEM (Li 2013) with default settings. This final set of 84,978 markers represents our high-confidence markers. Sample-specific allele frequencies were then calculated as their maximum-likelihood estimates given by the number of reads representing an allele at a given marker in a given sample divided by the total number of reads representing that marker within that sample.

### Genetic relationships

To assess the robustness of our allele frequency estimates across biological replicates, we calculated a genomic relationship matrix (VanRaden 2008; Goddard *et al.* 2011) with the expectation that samples originating from the same population (*i.e.*, O, H, or L) would be more closely related to one another than to samples from a different population, as determined by pairwise covariances. A tree of relatedness using Euclidean distances was also calculated. We calculated the genomic relationship matrix as:

$$G=\frac{WW^{T}}{m}$$ (1)

where *G* is a *n* × *n* genomic relationship matrix, *n* is the number of samples, *m* is the number of markers, and *W* is a *n* × *m* matrix of marker frequencies centered around their mean and scaled by the SD.

### Divergence scan

To evaluate divergence among populations for individual loci, we calculated $F_{ST}$ for each genome-wide marker for all pairwise combinations of the pre- and postselection populations using the heterozygosity-based formula from Nielsen and Slatkin (2013) but estimating heterozygosity in population *k* with allele frequency $p_{k}$ as $1-p_{k}^{4}-{\left(1-p_{k}\right)}^{4}.$

Another commonly used method to assess population structure from marker data is the use of principal component analysis (PCA) to decompose variation from correlated markers into uncorrelated dimensions (Patterson *et al.* 2006). Given the controlled nature of the original phenotypic selection experiment, we expected markers most strongly contributing to population subdivision to be under selection and thus trait related. To distinguish these candidates of selection, we followed the methodology of Luu *et al.* (2017) to identify markers excessively related to population structure in our experiment. Using our frequency estimates and the PCAdapt software (Luu *et al.* 2017), we generated sequencing read counts for every marker for 250 tetraploid individuals from the 12 pooled samples using the *read.pcadapt* function under pool-seq mode. These data were then used in conducting a PCA using the *pcadapt* function.

Using the first principal component, we calculated a z-score for the $j^{th}$ marker as described in Luu *et al.* (2017) for one principal component:

$$z_{j}={\hat{\beta }}_{j}\frac{\sqrt{\sum_{i=1}^{n}x_{i}^{2}}}{\sigma_{j}^{2}}$$ (2)

where ${\hat{\beta }}_{j}$ is the regression coefficient for the $j^{th}$ marker regressed on the first principal component, $\sigma^{2}$ is an estimate of the residual variance for the $j^{th}$ marker, and $x_{i}$ is the score on the first principal component for the $i^{th}$ individual. The z-scores were then used to calculate the test statistic, a robust Mahalanobis distance. Markers in the 0.1% tail of the test statistic were labeled as candidates for selection.

### *Medicago truncatula* genes near candidate markers

The genomic positions of candidate markers were queried against annotated *M. truncatula* genes (Mt4.0v2_genes_20140818_1100.gff3) to identify genes located near these markers. Based on findings of fast-decaying linkage disequilibrium in nondormant alfalfa germplasm (Sakiroglu *et al.* 2012; Li *et al.* 2014), we looked for genes located within 20 kb, on *M. truncatula*, of the candidate markers.

### Effective population size and drift simulations

We inferred genome-wide average effective population size (${\hat{N}}_{e}$) from the allele frequency data using a recently proposed estimator (Jónás *et al.* 2016) implemented in the R package *Nest* (https://github.com/ThomasTaus/Nest). This implementation extends previous methods of estimation from temporal data to pooled sequencing by correcting for the variance due to the inherent two-step sampling process. We estimated (${\hat{N}}_{e}$) from the O-H and the O-L population pairs using the appropriate frequency and coverage data as well as parameter values to represent 96 autotetraploid individuals per population separated by three generations under “Plan II” in *Nest* where sampled individuals are removed from the respective population.

After determining ${\hat{N}}_{e}$, we were able to simulate genetic drift to assess whether the observed changes in allele frequencies for our candidate markers could be accounted for by drift alone. For every candidate marker, 10,000 allele frequency trajectories were calculated to model drift in a Wright–Fisher population of size, in chromosomes, equal to the higher effective population size estimate from *Nest*. For every trajectory, *j* the number of alleles corresponding to the starting frequency in the base population were allowed to appear in a population of size ${\hat{N}}_{e}$ chromosomes. For every generation, sampling probabilities were calculated as the frequency of the allele in the prior generation. Binomial sampling was then conducted to generate counts in the current generation. Counts from the starting and the final generation were then binomially sampled, using a sample size of 384 chromosomes (96 autotetraploid individuals), and used in calculating the frequencies. Markers showing postselection frequency changes greater than could be described by a 99.9% confidence interval for genetic drift, defined using the simulated trajectories, were labeled as good candidates for selection.

### Data availability

All analyses, unless otherwise noted, were performed in (R Core Team 2015). Raw sequencing data were deposited to the Sequence Read Archive under accession PRJNA420993. Read count, frequency, and phenotypic data used for analysis are available for download from https://figshare.com/articles/CUF101-selmap/5266537. Scripts for analyses are available in a GitHub repository (https://github.com/grmunjal/singlepop).Please cite this paper if reusing any of these resources.

## Results and Discussion

### Selection for reduced autumn height in a nondormant background resulted in remarkable phenotypic and genotypic differentiation

**Figure 1:**
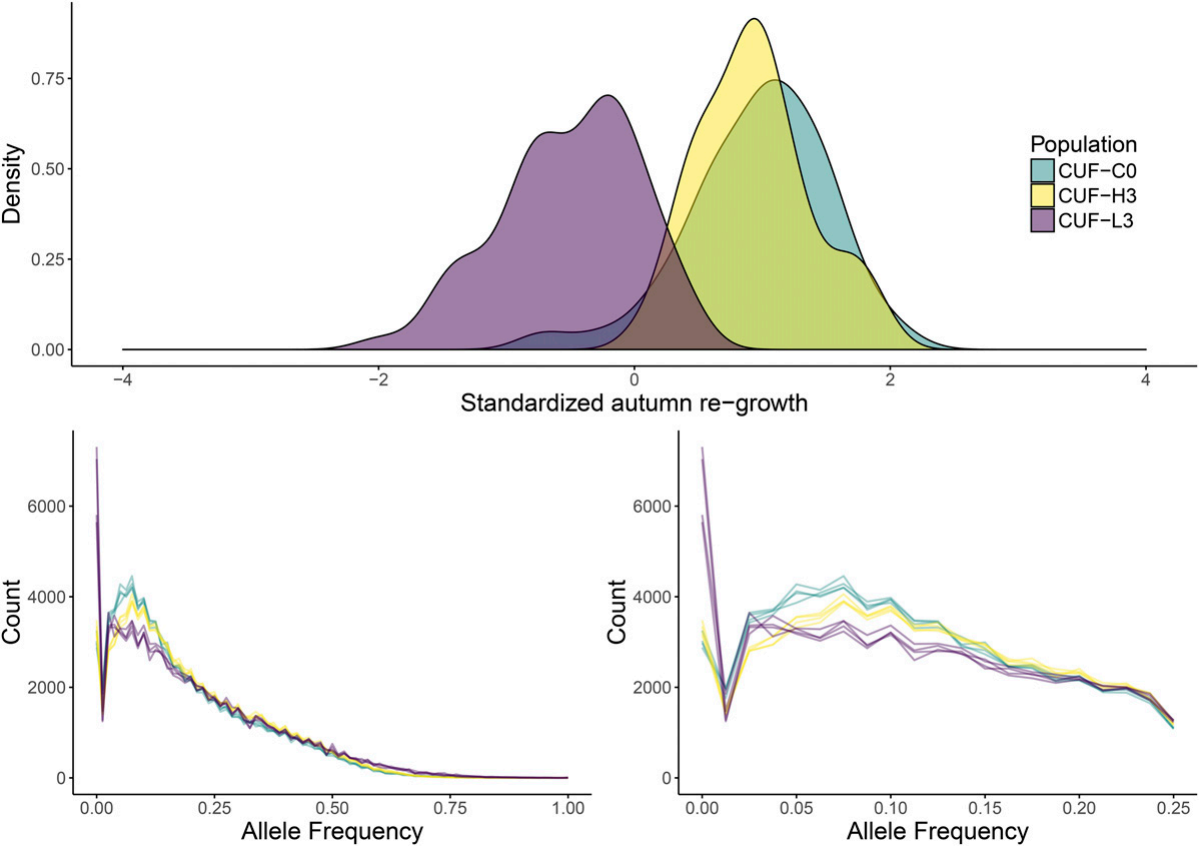
Top panel: distributions of autumn regrowth in the pre- and postselection populations evaluated in Davis, CA. Bottom panel: distribution of minor allele (across the experiment) frequencies of genome-wide markers. Each population is represented by four curves corresponding to each of four pools. The right panel is an expanded view of the leftmost section of the graph in the left panel.

In each of the three populations, some plants were either lost during transplanting and/or failed to establish in the field; consequently, the number of individuals available for phenotypic analyses was less than the number available for genotypic analyses, as tissue sampling was performed in the greenhouse prior to transplanting. The phenotypic variances of the preselection and postselection populations (Figure 1) were not different. As evidenced by the best linear unbiased estimators, selection was effective at reducing autumn height from 0.92 (*n* = 90) in CUF-O to −0.53 (*n* = 64) in CUF-L, but a contrasting response was not observed in CUF-H (0.93, *n* = 63). Failure to make genetic gain when selecting for less dormancy is curious, but it possibly suggests a lack of additive genetic variance for the trait in the starting population. Given that the starting population, CUF 101, is primarily nondormant, it may be approaching fixation for alleles underlying decreased dormancy and/or improved autumn growth. However, because a very nondormant germplasm Wadi Quarayat in Cunningham *et al.* (1998) responded to selection for taller plants, it is unlikely that a biological limit is curtailing gain in CUF 101.

Our estimates of the distributions of minor allele frequencies of genome-wide markers in each of the populations indicated that the phenotypic differentiation of the CUF-L population was accompanied by a strong genetic response (Figure 1). An excess of low-frequency variants postselection, as seen in CUF-L (Figure 1), is indicative of strong directional selection, as neutral loci closely linked to the loci under selection are swept to extreme frequencies (Smith and Haigh 1974). The genome-wide result of these changes was also apparent in branch lengths of a tree estimated from mean genome-wide $F_{ST}$ values (Supplemental Material, File S5), where the branch length of the shorter population (0.02; L in File S5), is nearly three times the branch lengths of the remaining populations (0.007 for O and 0.009 for H in File S5). Interestingly, a small enrichment of low-frequency alleles was also observed in the CUF-H population, indicative of a genetically differentiating force operating in this population that is apparently not related to dormancy.

### Allele frequency estimates are robust

Allele frequency estimates from pooled sequencing could be biased by two sources of variation: (1) sampling of chromosomes during pooling, and (2) sampling of reads in sequencing. The robustness of maximum-likelihood allele frequency estimates calculated from pooled sequencing to these sources of error is a topic of debate in the literature (Zhu *et al.* 2012; Gautier *et al.* 2013; Rellstab *et al.* 2013; Lynch *et al.* 2014). Here, we sought to assess whether allele frequency estimates calculated using our population-level filtering criteria based on 96 plants were robust by assessing frequencies within subsamples of the overall population, an empirical approach essentially similar to that of Byrne *et al.* (2013), who investigated the reliability of frequency estimates across sampling replicates.

**Figure 2:**
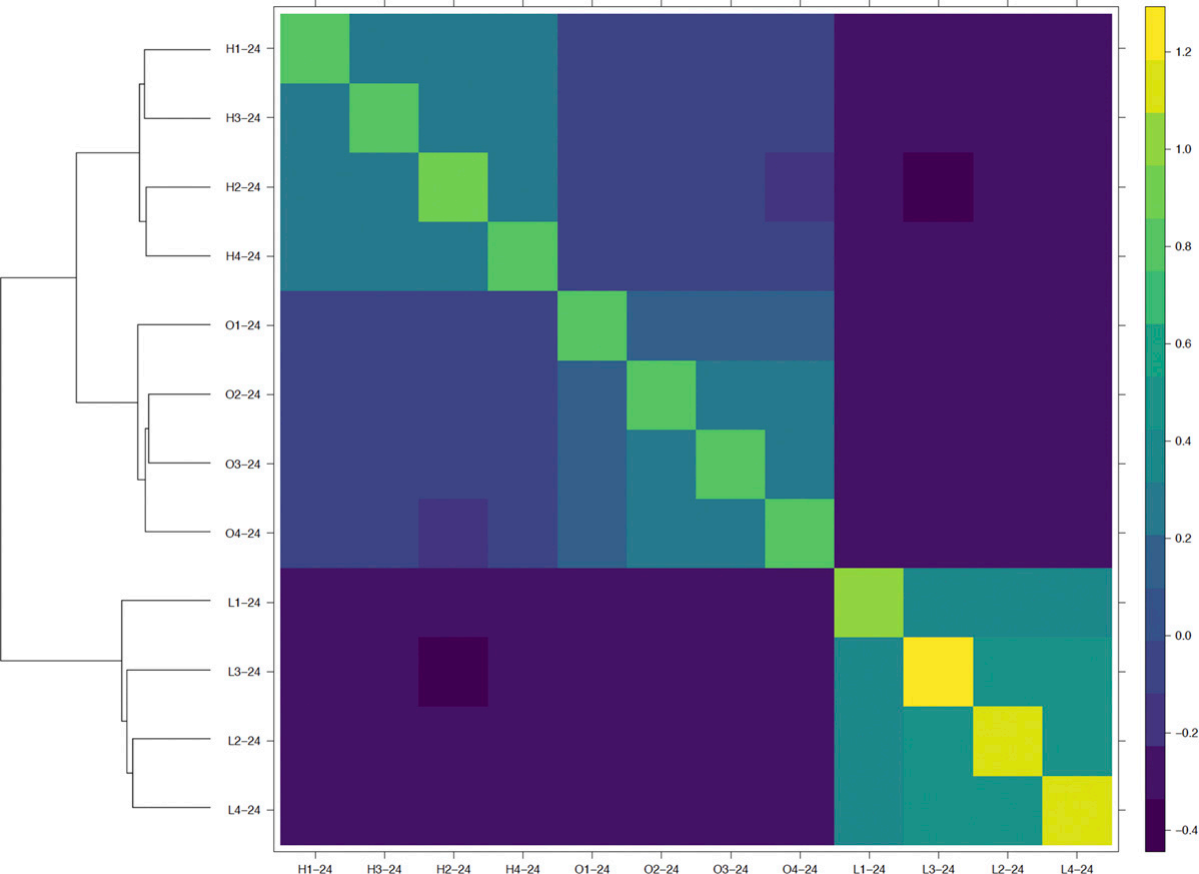
Heat map of a genomic relationship matrix constructed using 24 plant sampling-level pools. Labels are named by the convention “population”-“number of plants.” Dendrogram represents tree based on Euclidean distance between populations.

Covariance between samples is directly proportional to the degree of relatedness, while covariance of a sample with itself is inversely proportional to the amount of heterozygosity. A genomic relationship matrix calculated using sampling-level pools indicated that each of the samples was genetically closer to other samples from the same population than to samples from another population, showing that our frequency estimation criteria were able to retain population-specific signals (Figure 2). The observed genomic relationships between the populations parallel the phenotypic observations, as samples from the CUF-L population are estimated to be much more genotypically removed from the starting population than samples from the less-dormant population, as indicated by the off-diagonal covariance elements of the matrix. Inspection of the variance terms (*i.e.*, the diagonal elements) highlights that, across populations, there is more homozygosity (or alternatively less heterozygosity) in the CUF-L population. These findings are in agreement with the expectations for directional selection and reflect the observation of more alleles at or close to fixation in CUF-L (Figure 1).

### Frequency shifts suggest directional selection

**Figure 3:**
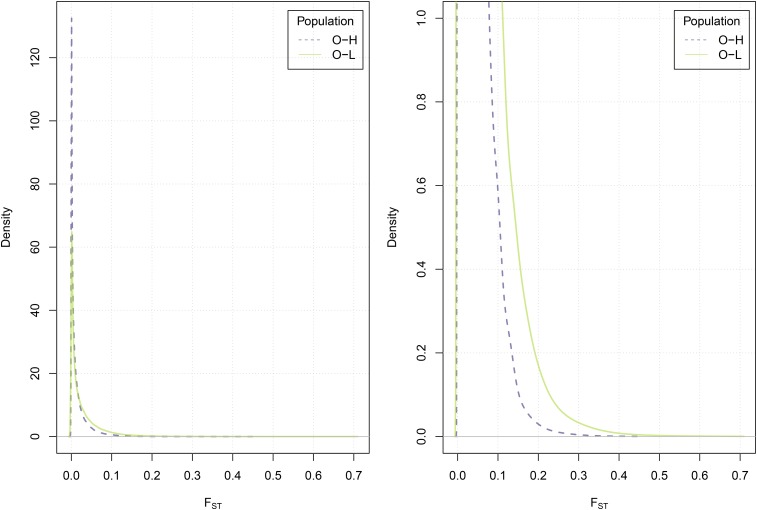
Distributions of $F_{ST}$ between combinations of the pre- and postselection populations. Right panel is a zoomed-in version of the tails of the distributions. Elevated levels of the statistic are observed in the population pair (O-L) showing selection response in comparison with the pair showing no response (O-H).

Phenotypically, the CUF-O and CUF-H populations were not different. In order to assess genomic divergence, we used two methods, one based on $F_{ST}$ (Beissinger *et al.* 2014) and the other on PCA (Duforet-Frebourg *et al.* 2015; Luu *et al.* 2017). Mean $F_{ST}$ values across all loci showed that CUF-O|CUF-H had a small value (0.0162, 99% bootstrap confidence interval 0.0159–0.0164) compared with CUF-O|CUF-L (0.0294, 99% bootstrap confidence interval 0.0290–0.0298]) (Figure 3), reflecting the phenotypic separation we observed in the field.

For the PCA method, we used a scree plot (File S1) to show that the first two principal components were most related to the population structure observed in the selection experiment. PC1 effectively differentiated CUF-L from CUF-O and CUF-H, with PC2 separating CUF-O and CUF-H. Since the latter two populations were not phenotypically different, we expected markers most strongly separating the CUF-L population from the remaining two to be dormancy related (File S2). Consequently, we only used the first principal component for our subsequent differentiation analysis.

**Figure 4:**
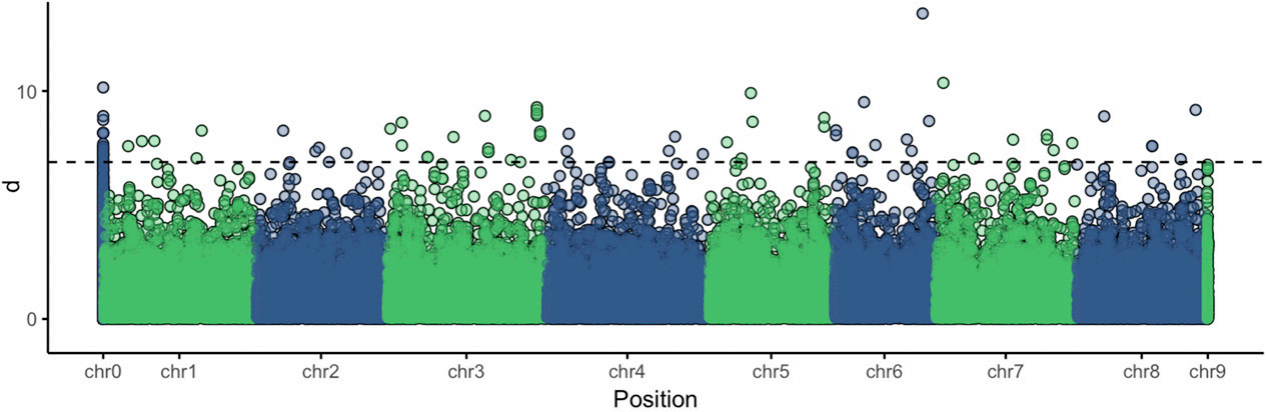
Manhattan plot of *d* (Mahalanobis distance) calculated using PC1 scores. Mapping positions are based on alignment to the *M. truncatula* reference genome. The synthetic chromosomes “chr0” and “chr9” represent markers that do not align to *M. truncatula* chromosomes but have valid alignments to both CADL and *M. truncatula* scaffolds (“chr9”) or CADL scaffolds only (“chr0”).

Based on z-scores computed for each marker locus, we identified 85 loci as potentially under selection because they exceeded a 99.9% empirical threshold (Figure 4). Of these 85 loci, 79 were also in the 1% tail of CUF-O|CUF-L $F_{ST}$ values, providing additional evidence that these loci could be associated with dormancy. Of the 85 loci, 22 had no valid alignments to *M. truncatula*, suggesting that they are alfalfa specific, as they did align to the CADL scaffolds. Positions and annotations of *M. truncatula* genes within 20 kb of the remaining 63 loci can be found in File S3. Some of the genes in this small set have been previously implicated in desiccation or cold tolerance, including expansins, late embryogenesis abundant proteins, dehydrins, and heat shock proteins (Oliver 2007; Boudet *et al.* 2006; Verdier *et al.* 2013). That our results include genes that could be expected to be identified shows that selection mapping is a powerful way to identify loci that have a role in autumn dormancy. Although there is considerable evidence of high genome synteny between *M. truncatula* and *M. sativa* (Li *et al.* 2014), the relative significance of the *M. truncatula* genes identified here on the overall dormancy phenotype is not clear. Unlike alfalfa, *M. truncatula* is an annual species that grows in a Mediterranean habitat, where autumn dormancy would be neither needed nor expressed. Obviously, the loci showing frequency changes that were unique to alfalfa Figure 4 may be expected to be significant, given the biology of perennial alfalfa.

### Effective population size estimates agree with expectations from known origins of CUF 101

The ancestry of CUF 101 traces back to seed bulked from 91 parent plants in a field in El Centro, California (http://www.naaic.org/varietyaps/CUF101.html). These plants were selected for their superior disease resistance. The vast majority (89 plants) of these plants were derived, directly or indirectly, from “UC Cargo” (https://www.naaic.org/varietyaps/UCcargo.html), most of whose parents trace to “UC Salton” (https://www.naaic.org/varietyaps/UCsalton.html), which basically descends from a single genetically diverse germplasm. Population genetic theory dictates that the effective population size is usually smaller than the census size. In the presence of a selective force, as is the case for CUF 101, we expect the effective size to be even smaller.

Using a Wright–Fisher model-based approach, we estimated the effective size for CUF 101 to be ∼15 autotetraploid individuals from the CUF-O|CUF-H population pair and ∼7 autotetraploid individuals from the CUF-O|CUF-L population pair. The former is likely a more realistic approximation as, unlike the latter, it is not confounded by a strong selection response. We justify using a Wright–Fisher model-based approach for inferring effective population size from autotetraploid frequency data based on recent extensions of Kingman’s coalescent (Kingman 1982) to autotetraploids that demonstrate its robustness to tetrasomic inheritance (Arnold *et al.* 2012) under the assumption that double-reduction and selfing are absent or rare.

### Drift simulations

The loci identified from our PCA may have differentiated as a result of directional selection, and hence could be dormancy associated, or they could differ owing to genetic drift. To test whether frequency changes for these loci could be explained by drift, we conducted simulations of genetic drift for each of the candidate loci (File S4). In the shorter (L) population, all 85 markers were found to have frequency changes greater than the calculated confidence intervals. In the taller (H) population, 10 out of the 85 markers were found to be have frequency changes greater than the calculated confidence intervals; however, these changes had similar directionality to the shorter population, indicating that although directional selection for autumn dormancy was a pervasive force in selection for more dormancy, a genetic influence other than selection for the focal trait and genetic drift may have been involved as well. We hypothesize that these 10 loci experienced a selective pressure separate from that intentionally applied based on autumn plant height. One explanation could be related to survival; Figure 1 in Cunningham *et al.* (1998) shows increased survival for both CUF-H (7%) and CUF-L (93%) in comparison with CUF-O, a result not commented upon in that paper. Further, it is also conceivable that differential flowering time within the selected progeny during intercrossing in the greenhouse, as described in Cunningham *et al.* (1998), could have resulted in assortative mating and consequent allele frequency shifts unrelated to selection for dormancy. Additionally, three markers appeared to be fixed in the starting and less-dormant populations but nearing fixation in the opposite direction in the shorter population. Given that allele frequency estimates are simple ratios of read counts, it is likely that these markers have extremely low frequency in the former two populations.

### Future directions

Selection mapping offers the unique opportunity to identify variants affecting a trait by following their accumulation over generations of selection. Further, population-based pooled sequencing makes the costs of experiments like this easily within reach of most laboratories. The plethora of recurrently selected populations available to the plant science community provides a unique resource on which to apply selection mapping to conduct genetic studies of various traits. Here, we used populations from one experiment to calculate population-specific allele frequency estimates and scan the genome for loci controlling autumn dormancy in alfalfa. We are currently evaluating similar changes in five other alfalfa populations divergently selected for autumn dormancy, giving us further power to detect changes across genetic backgrounds as well as to identify population-specific changes. By combining these results with those found in other experiments using biparental or GWAS populations, transcriptome analyses, or others, we will be able understand this critically important alfalfa trait.

## Supplementary Material

Supplemental material is available online at www.g3journal.org/lookup/suppl/doi:10.1534/g3.117.300099/-/DC1.
