# Supplementary figures and images for: Selection Mapping Identifies Loci Underpinning Autumn Dormancy in Alfalfa (Medicago sativa)
Source: G3 (Bethesda). 2017 Dec 18;8(2):461–8. doi: 10.1534/g3.117.300099 (PMC5919736; doi:10.1534/g3.117.300099)

Scree Plot – K = 20

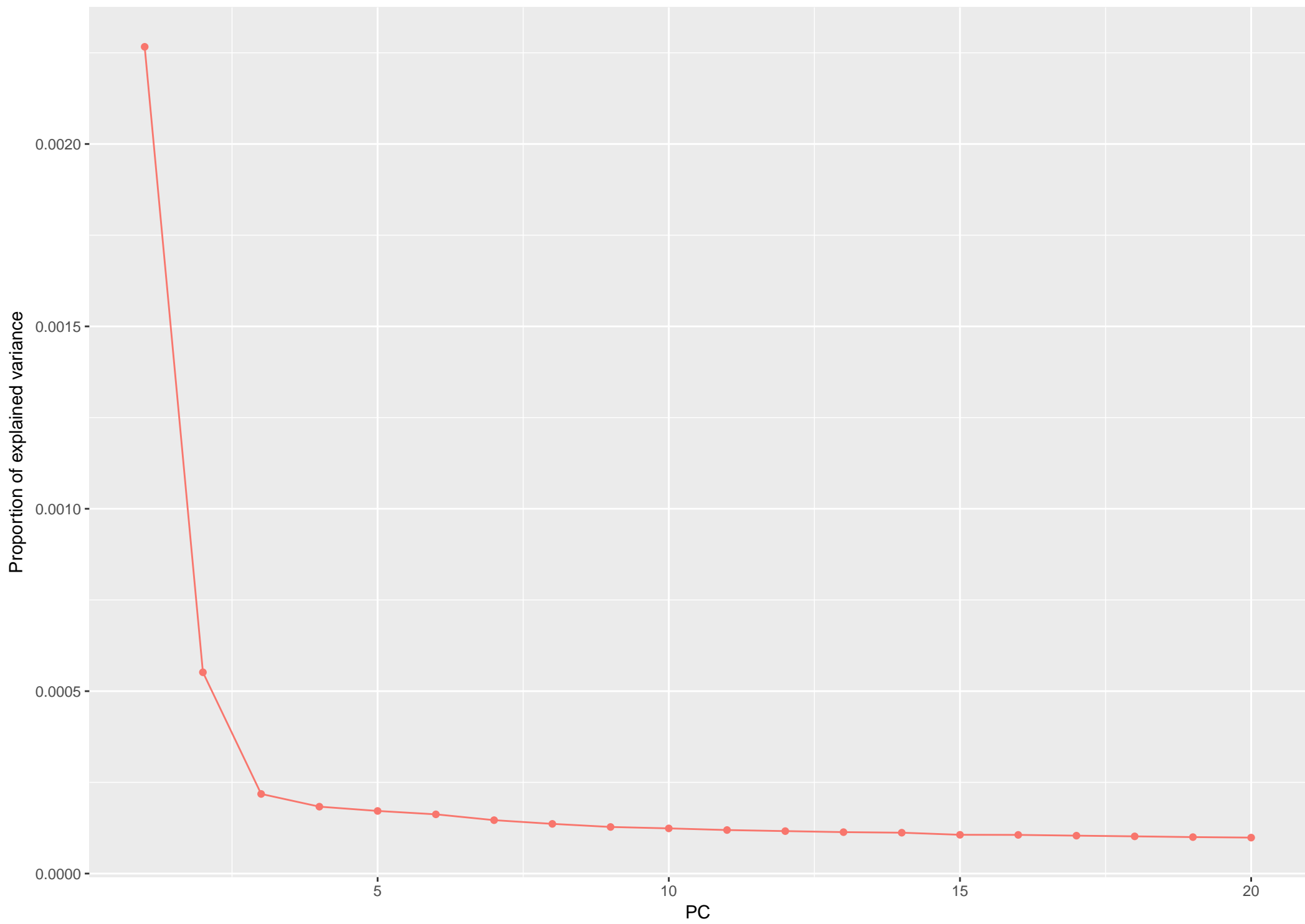

Projection onto PC1 and PC2

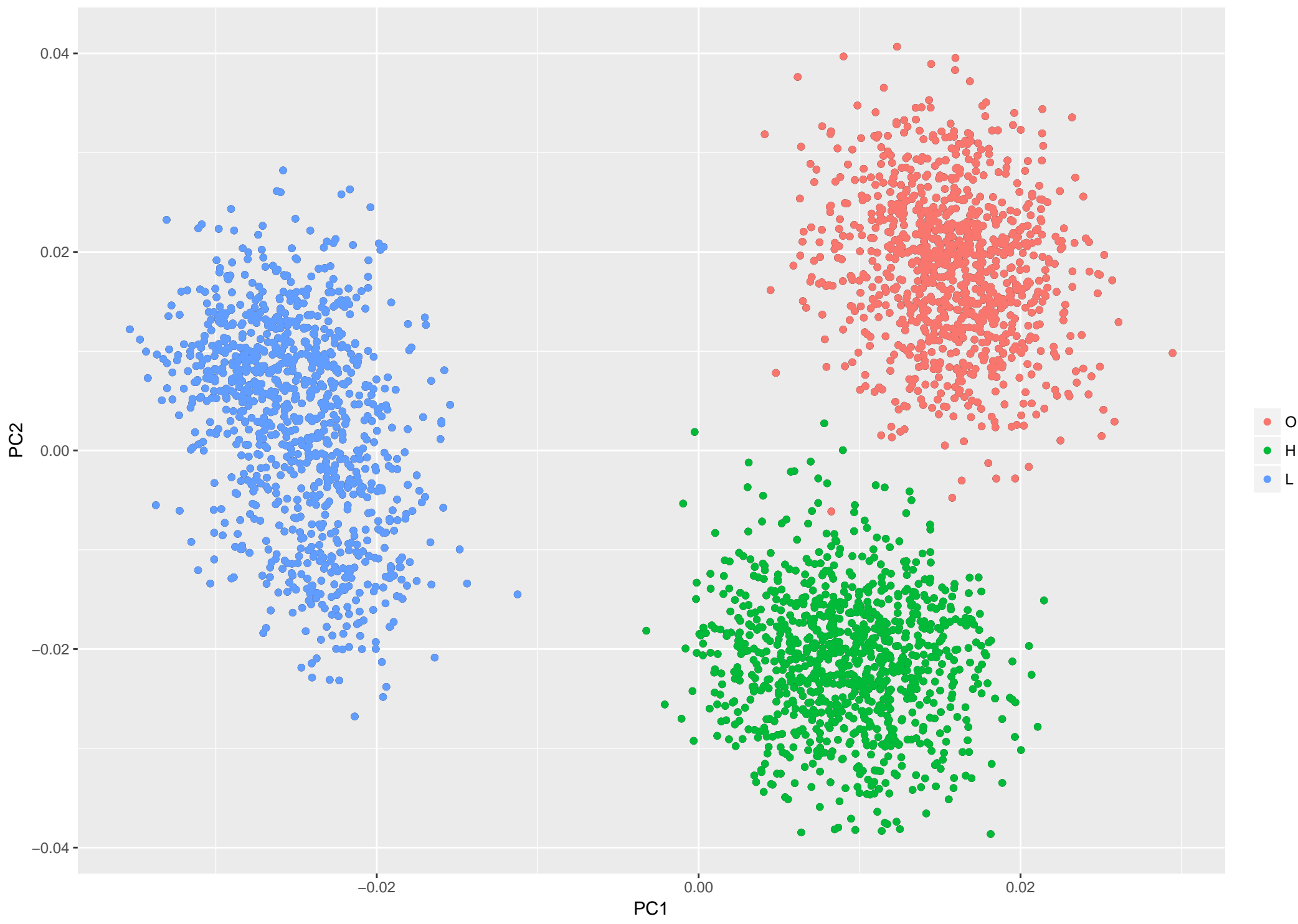

Supplement: Supplementary file 1 [file 461FileS1.pdf]

m = 84978 markers

PC2

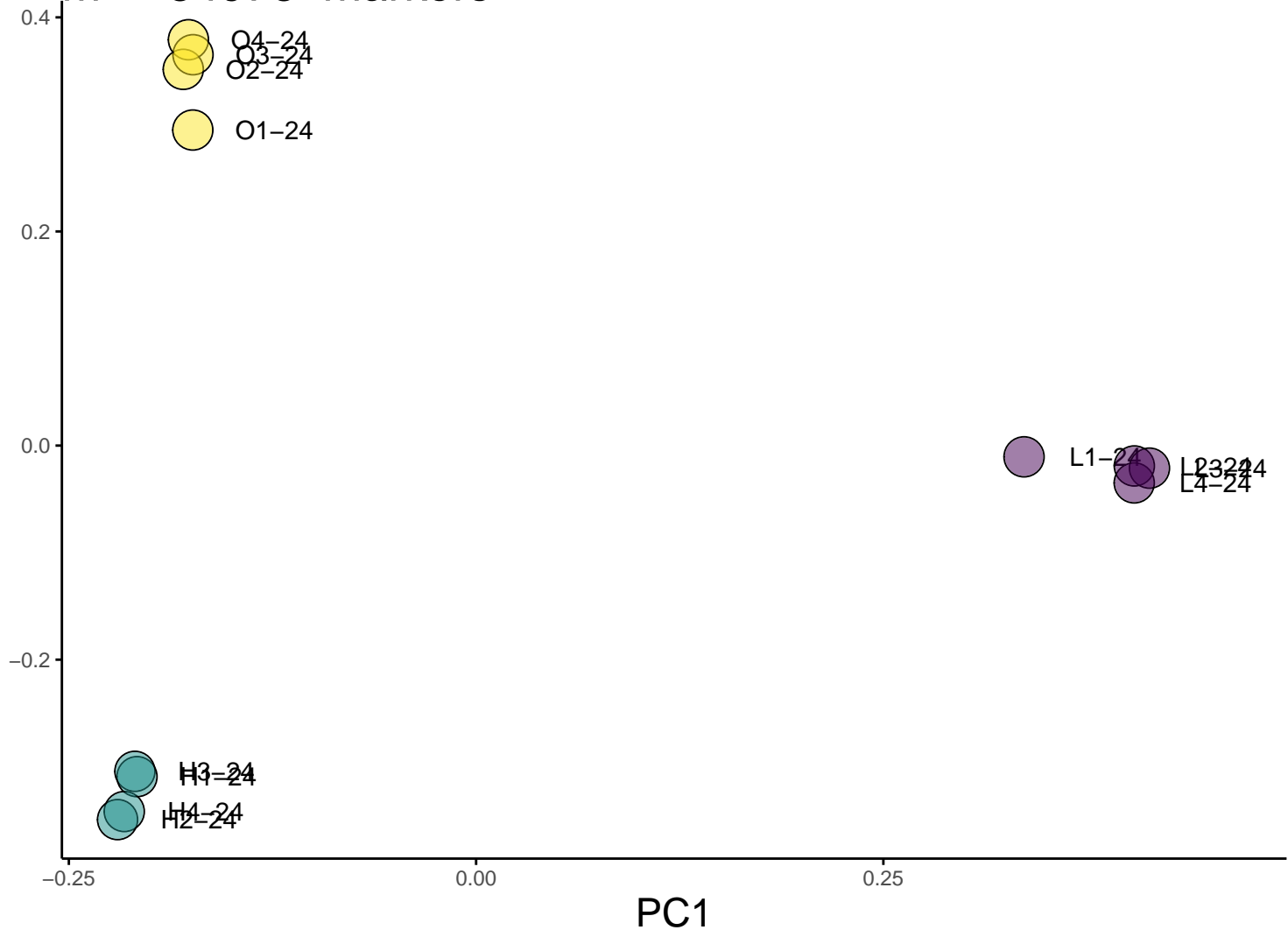

Supplement: Supplementary file 2 [file 461FileS2.pdf]

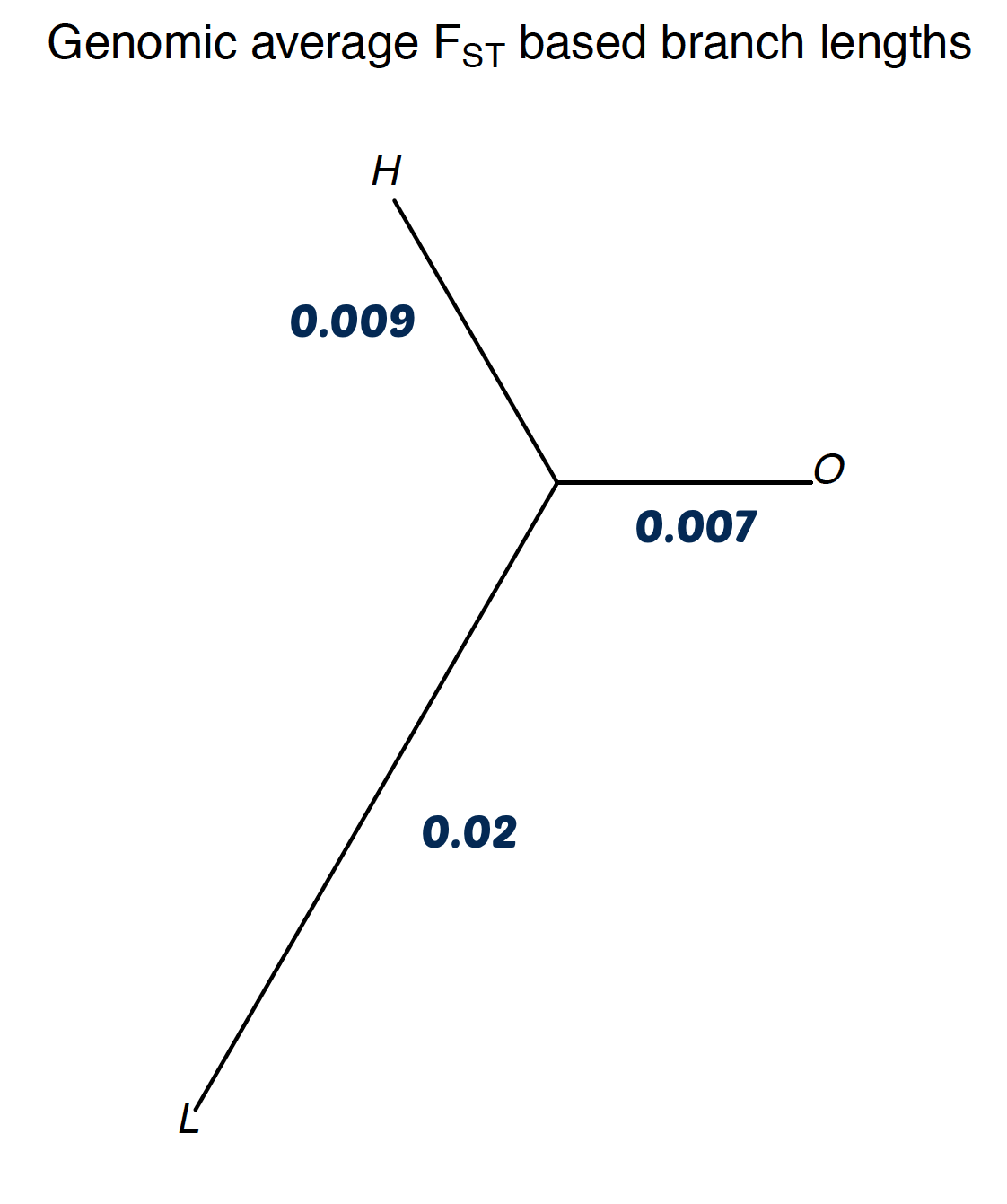

Supplement: Supplementary file 5 [file 461FileS5.png]
