# Supplementary material for: Selection Mapping Identifies Loci Underpinning Autumn Dormancy in Alfalfa (Medicago sativa)
Source: G3 (Bethesda). 2017 Dec 18;8(2):461–8. doi: 10.1534/g3.117.300099 (PMC5919736; doi:10.1534/g3.117.300099)
Supplement: Supplementary file 4 [file 461FileS4.pdf]

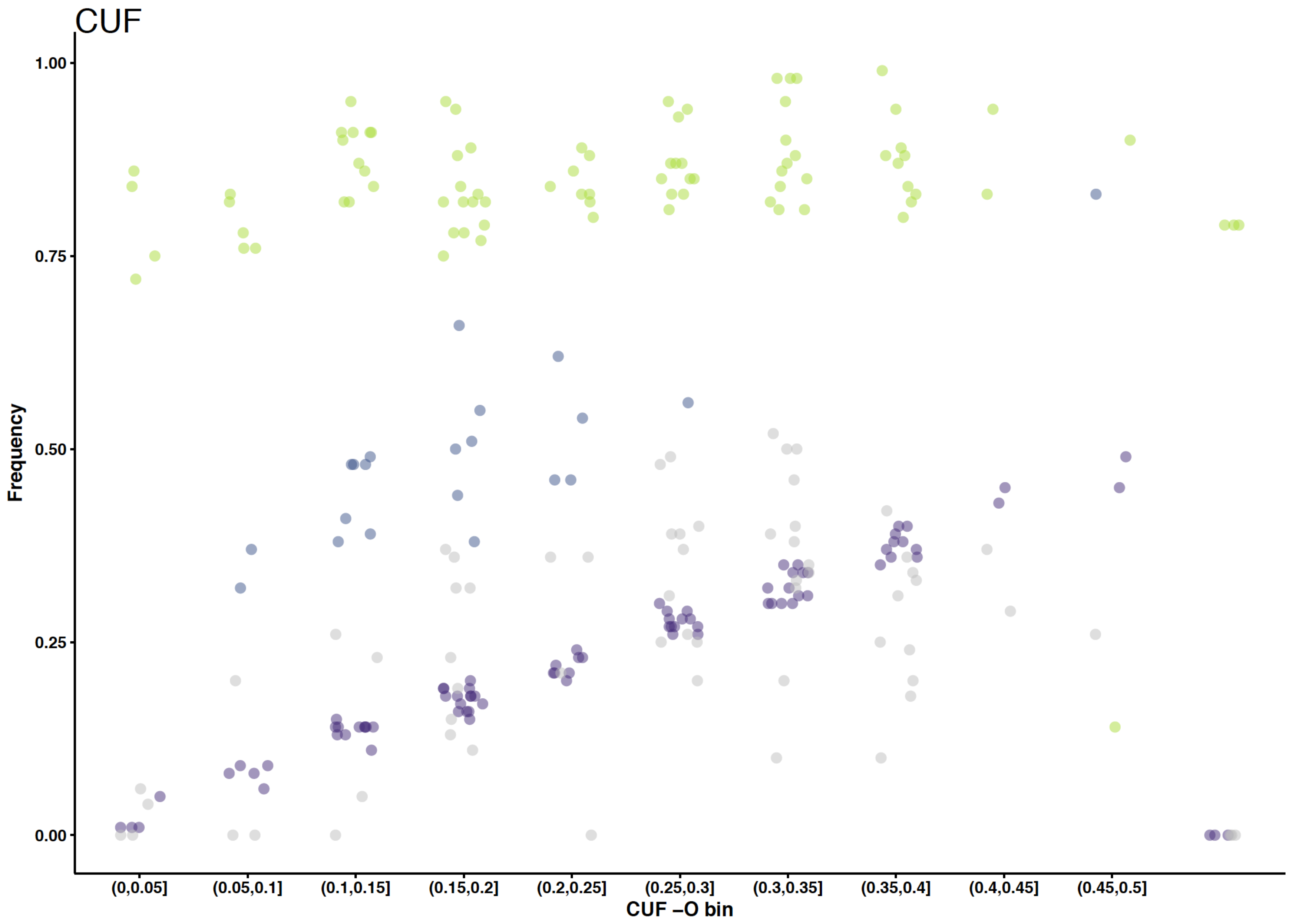

Pre- and post-selection frequencies of candidate markers binned by their frequencies in the starting (CUF-O) population. Purple, blue, and green dots represent the CUF-O, CUF-H, and CUF-L populations respectively. Grey dots represent CUF-H and CUF-L markers within a 99% drift confidence interval.
